# Comparison of the optimal and suboptimal quantity of mitotype libraries using next-generation sequencing

**DOI:** 10.1007/s00414-023-03099-7

**Published:** 2023-09-30

**Authors:** Marcel Obal, Tomaž Zupanc, Irena Zupanič Pajnič

**Affiliations:** Institute of Forensic Medicine, Faculty of Medicine, University of Ljubljana, Korytkova 2, 1000 Ljubljana, Slovenia

**Keywords:** Mitogenome, Second World War, Haplotype, Next-generation sequencing, Library input

## Abstract

Optimizing analysis parameters and sample input is crucial in forensic genetics methods to generate reliable results, and even more so when working with muti-copy mitochondrial DNA (mtDNA) and low-quality samples. This study compared mitotypes based on next-generation sequencing (NGS) results derived from the same samples at two different sequencing library concentrations—30 pM and 0.3 pM. Thirty femur samples from the Second World War were used as a model for poorly preserved DNA. Quantitative PCR (qPCR) method targeting 113 bp long fragment was employed to assess the quantity of mitogenomes. HID Ion Chef™ Instrument with Precision ID mtDNA Control Region Panel was used for library preparation and templating. Sequencing was performed with Ion GeneStudio™ S5 System. Reference haplotypes were determined from sequencing samples at 30 pM library input. Haplotypes were compared between optimal (30 pM) and suboptimal (0.3 pM) library inputs. Often the difference in haplotypes was length heteroplasmy, which in line with other studies shows that this type of variant is not reliable for interpretation in forensics. Excluding length variants at positions 573, 309, and 16,193, 56.7% of the samples matched, and in two samples, no sequence was obtained at suboptimal library input. The rest of the samples differed between optimal and suboptimal library input. To conclude, genotyping and analyzing low-quantity libraries derived from low-quality aged skeletonized human remains therefore must be done with caution in forensic genetics casework.

**Supplementary Information:**

The online version contains supplementary material available at 10.1007/s00414-023-03099-7.

## Introduction

Mitochondrial DNA (mtDNA) has been of utmost importance and interest in routine casework in forensic genetics as well as in research, even though it is lacking some forensically desirable qualities, such as sufficiently high discriminatory power. MtDNA has unique properties—namely, maternal inheritance and a closed circular shape—resulting in lower susceptibility to environmental factors and multi-copy presence in a single cell [1]. All these qualities result in a higher copy number per cell and more preserved genome molecules in comparison to nuclear DNA (nDNA), both proving to be beneficial. Because of this, lower-quality samples that are challenging to investigate are usually analyzed using mtDNA [2]. The non-coding segment of mtDNA called the control region—with its considerably higher degree of variability because of its higher mutation rate—makes this section of the mitogenome favorable in the context of forensic genetics analyses, allowing a certain level of distinction, for example, between maternal lineages or populations [3]. It can be exploited in cases involving missing persons and crime cases [2], ancient DNA (aDNA) analyses [4], historical cases in which potential relatives through maternal lineage are sought [5, 6], or for predicting mtDNA haplogroup and placement into a genetic population to determine biogeographical origin [7].

Expeditious genotyping advancements in recent years have shifted mtDNA analyses from classic Sanger to next generation sequencing methods, the latter introducing benefits such as considerably higher throughputs, less time to be consumed for analysis, and standardization and automatization possibilities [8, 9]. However, with new methods arise new considerations about sample inputs and parameters that need to be addressed in order to provide the most reliable results.

First parameter to consider is mtDNA copy number, which can vary greatly between tissues [10]. The next parameter to consider is the quantity of sequencing library used for pooling, which can affect the genotyping results. If the input is too high, it may overload the platform; if the input is too low, there might not be optimal performance utilization. If the pool contains non-equimolar proportions of libraries, coverage may vary between samples, making genotyping results potentially unreliable [11].

This study compares and examines two sets of NGS Precision ID mtDNA Control Region Panel (TFS) genotyping data results by analyzing mtDNA isolated from poorly preserved bone samples from Second World War. For each sample 5000 copies were used for automated library preparation, as it was suggested to use between 1000 and 5000 copies as optimal mtDNA input (Parson W. personal correspondence, MtDNA Analysis and interpretation using EMPOP, ISFG pre-congress workshop, Prague, September 9th 2019). This was done to ensure the optimal sequencing results when defining reference haplotypes (with optimal library input) that were used for comparison with haplotypes determined from suboptimal library input. Two different library concentrations—one normalized to optimal (30 pM), and another diluted (100-fold) to suboptimal (0.3 pM) quantity—were used. The aim was to explore whether low-concentration libraries that can derive from low-quantity and low-quality samples, are able to provide reliable haplotypes to be used in forensic casework.

This study was approved by the Slovenian Medical Ethics Committee, approval number 0120–22/2017/3.

## Materials and methods

### Samples

Parts of skeletal elements—namely, the diaphyses of femurs of thirty victims from a Second World War mass grave, which was described in extensive detail in previously published research [12]—were chosen to be used as examples of challenging forensic samples.

### Contamination safeguard procedures

The tools used for sample preparation were cleaned with 6% sodium hypochlorite, rinsed with bidistilled water, washed with 80% ethanol, and subsequently sterilized. After the tools, reagents, and laboratory plastics had been sterilized, they were all placed under UV light in the last step of contamination prevention. Disposable gloves, caps, and gowns were used to prevent contamination by modern DNA from personnel.

### Sample preparation and DNA extraction

Sample preparation was performed in a room specifically intended for working with skeletonized human remains. To remove any potential surface contaminants, the outer layer was removed with burrs and separating discs attached to a rotary tool (Schick, Schemmerhofen, Germany); the process was carried out in a fume hood (Iskra Pio, Šentjernej, Slovenia). Cleaning was then further performed with detergent (5% Alconox; Sigma-Aldrich, St. Louis, MO, USA), followed by rinsing with bidistilled water (Millipore, Darmstadt, Germany) and washing with ethanol (80%; Fisher Scientific, Loughborough, UK), and bone pieces were left to dry overnight. Once dried and broken into smaller pieces, the bone was pulverized into a fine powder with a Bead Beater MillMix 20 homogenizer (Tehtnica, Domel, Železniki, Slovenia) at an oscillation frequency of 30 Hz for 1 min in metal grinding jars that were pre-cooled with liquid nitrogen. The bone powder was then weighed, and 0.5 g was used for an overnight incubation with 10 ml of ethylenediaminetetraacetic acid (EDTA; Promega, Madison, WI, USA) to achieve complete demineralization. The optimized process that was followed is described in greater detail in a previous study [13]. Lysates were purified in a Biorobot EZ1 device (Qiagen, Hilden, Germany) with the EZ1 DNA Investigator Kit (Qiagen) and trace protocol chosen to elute 50 µl of DNA in a Tris EDTA buffer solution as per the manufacturer’s instructions. Together with bone samples, extraction negative control (ENC) was included to check for possible contamination.

### DNA quantification

The copy number of mtDNA was assessed by employing an in-house qPCR method previously published by Alonso et al. [14] using a 620 bp long fragment as a standard and by quantifying a 113 bp long fragment of mitogenome defined by custom-ordered primers (Applied Biosystems (AB), Renfrewshire, UK), using the QuantStudio™ 5 Real-Time PCR System (TFS) and Design and Analysis Software v1.5.2 (TFS). All samples were quantified at least in duplicate. As an alternative to 1X TaqMan Universal PCR Master Mix (AB), which was used in the initial study [14] but is not available anymore, a newer commercially available alternative was used: TaqMan™ Universal Master Mix II with Uracil-N-glycosylase (TFS). All samples had the mitogenome concentration normalized to 5000 copies/µl before automated library preparation in order to ensure same conditions prior to library preparation.

### Automated library preparation and templating

Automated combined library preparation was carried out on an HID Ion Chef™ Instrument (TFS) with the Precision ID DL8 Kit™ (TFS) and Precision ID mtDNA Control Region Panel (TFS), as recommended by the manufacturer [15]. Five thousand copies of mitogenome per sample were used. The number of primer pools was set to 2, the cycle number to 22 cycles, and anneal and extension time to 4 min. Each combined library was quantified in duplicate with the Ion Library TaqMan™ Quantitation Kit (TFS) on a QuantStudio™ 5 Real-Time PCR System (TFS) per the manufacturer’s guidelines [15], and raw data were interpreted with Design and Analysis Software v1.5.2 (TFS). The equimolar amounts required for super-pooling libraries were calculated as recommended by the manufacturer. Prior to templating, two sets of combined libraries were made: one with equimolar amounts as calculated per the manufacturer’s recommendations (30 pM), and another one with a 100-fold dilution (0.3 pM) of every library. Each library was then templated onto an Ion 530™ Chip (TFS).

Templating to an Ion 530™ Chip (TFS) was fully automated and carried out on an Ion Chef™ Instrument (TFS), using Ion S5™ Precision ID Chef Supplies, Ion S5™ Precision ID Chef Reagents, and Ion S5™ Precision ID Chef Solutions (TFS), and the libraries were joined into one combined pool, following the manufacturer’s manual [15].

### Next-generation sequencing and data analysis

Following the manufacturer’s recommendations [15], the Ion GeneStudio™ S5 System (TFS) together with Ion S5™ Precision ID Sequencing Reagents and Ion S5™ Precision ID Sequencing Solutions (TFS) were used to generate raw data for mtDNA sequencing.

Primary analysis of raw data, including sequence alignment to rCRS, and variant calling was performed with Ion Torrent™ Suite 5.10.1 (TFS) software and HID Genotyper 2.2 and Coverage Analysis (v5.10.0.3) plugins. Analysis settings were set to default.

Secondary data analysis was carried out with Converge™ Software v2.3 (TFS).

Length variants at positions 309, 573, and 16,193 (and the respective coinciding length heteroplasmy) were excluded as a parameter for differentiation when observing differences between reference haplotypes and suboptimal library input haplotypes.

## Results

### qPCR

MtDNA copy number per µl was based on quantification of a 113 bp long fragment. Sample 22 (S22) had the lowest copy number (5547), whereas sample S27 contained the most copies (97,838) (see Supplementary Material 1—SM 1).

### Next-generation sequencing

Library pool (LP) quantifications were as follows: LP_1_ = 146.178 pM; LP_2_ = 89.791 pM; LP_3_ = 57.396 pM; LP_4_ = 69.803 pM. These were then normalized to 30 pM and used as is for one combined library pool to determine reference haplotypes, and 100-fold dilutions (0.3 pM) were used for the second combined library pool.

#### Chip with optimal (30 pM) library input—reference haplotypes

**Fig. 1:**
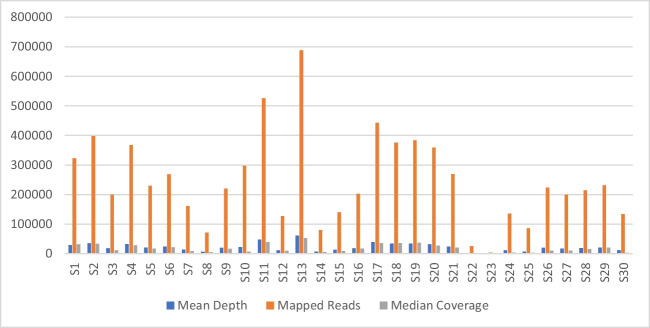
Mean depth, mapped reads, and median coverage for each sample at optimal library input

Ion Sphere™ Particles (ISP) loading to the chip was 85%, out of which 26.6% or 8,309,597 usable reads were in the final library. The mean read length was 118 bp, and the median read length was 121 bp. Mapped reads ranged from 3213 (S23) to 688,016 (S13). Mean coverage depth ranged from 23 as seen in sample S23, to 61,199 as seen in sample S13. (Fig. 1; also see SM 2).

#### Chip with suboptimal (0.3 pM) library input

**Fig. 2:**
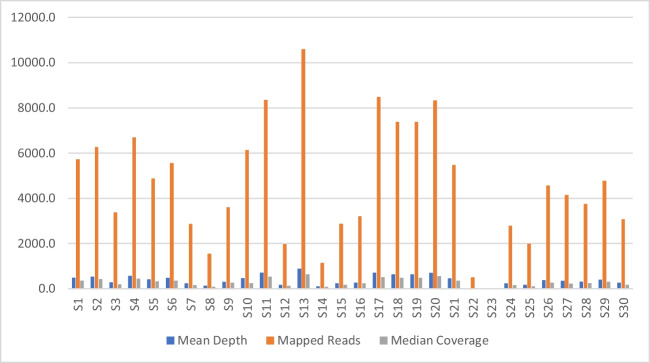
Mean depth, mapped reads, and median coverage for each sample for suboptimal library input

ISP loading to the chip was 4%, and out of loaded ISPs, there were 18.4% or 147,576 usable reads that the final library was compiled from. The mean read length was 112 bp, and the median read length was 116 bp. Mapped reads ranged from 25 (S23) to 10,793 (S13). Mean coverage depth ranged from 0.264 as seen in sample S23 to 888 as seen in sample S13 (Fig. 2; also see SM 2).

### Haplotypes

All haplotypes (with frequencies, strand biases, variant coverages, and scores) for optimal as well as suboptimal library input for respective samples are shown in SM 3.

Seventeen samples (S4, S5, S6, S8, S9, S11, S14, S15, S16, S17, S19, S21, S25, S26, S27, S28, and S29) showed no differences in haplotypes between optimal (reference haplotype) and suboptimal library input when comparing sequences (SM 3, gray-colored tabs). In some of these samples, length variants at positions 309, 573, and 16,193 (and a possible length heteroplasmy at those positions) were observed (as shown in SM 3) and were not included in haplotype comparison. In cases where those variants were the only difference between the haplotypes, such haplotypes were consequently characterized as having “no difference” between them.

Samples S22 and S23 had only reference haplotypes determined, and no results were obtained from suboptimal library input (SM 3, orange-colored tabs).

In samples S1, S2, S10, S12, some variants present at optimal library input, which was a reference haplotype, were not detected at suboptimal library input (SM 3, blue-colored tabs).

Samples S3, S7, S13, S18, S20, S24, and S30 presented differences between reference haplotypes and suboptimal library input (SM 3, purple-colored tabs).

In SM 3, frequencies highlighted with green color indicate that 90% or more reads agree with the variant call, and frequencies highlighted with yellow color indicate that less than 90% of reads agree with the variant call (see SM 3). Variant strand biases highlighted with green color indicate that forward and reverse reads that make up a variant call are distributed evenly. Yellow color indicated an uneven distribution of reads that make up a variant call between strands. Bright red color indicates a strong one-sided strand direction of reads that make up a variant call (see SM 3).

## Discussion

Seventeen out of 30 analyzed samples (56.7%) were matching between reference haplotype (30 pM library input) and suboptimal library input (0.3 pM). The latter takes into consideration that some variants (e.g., length variants at positions 309, 573, and 16,193 and a possible length heteroplasmy at those positions) were excluded from haplotype comparison, as also seen in other studies [16, 17], because these variants, especially in a combination with technology used, are not reliable to be used for haplotype comparison in forensics, and that in one case, there was a variant called that was labelled “degraded.”

When comparing variants in this study, length variants at positions 309, 573, and 16,193 (and affiliated length heteroplasmy) were often the main difference that set the haplotypes apart between reference and low library quantity input. Nevertheless, these variants were not always specific to either optimal nor sub-optimal inputs as they arise in both cases, so a definite conclusion cannot be made, that these differences are a direct result of different library quantity inputs. It does, however, fortify the recommendations of some studies, that when mitogenome sequences generated with Ion Torrent™ technology are analyzed, length heteroplasmies are better to be excluded from haplotype comparison, especially in forensic genetics. Ion Torrent™ technology is unable to provide high enough base number resolution when there is a sequence of multiple consecutive bases of the same type (e.g., length heteroplasmy such as C-stretch), and therefore such patterns should be interpreted with caution or excluded from analyses when comparing to a reference haplotype, as they were in our case [18, 19].

In samples S22 and S23, only reference haplotypes were possible to generate. Variant coverages were very low in both cases, being as low as 24 (variant 73G in S23). In contrast, for reference haplotypes in other samples median coverage spanned to several thousand. Since such metrics occurred when there was only a reference haplotype determined, this might need to be interpreted with caution.

Differences in variant calls in S7 (16183 del, 16186 T, and 16188 T in reference haplotype, and 16183C, 16185 T, and 16187 del at suboptimal input) might point towards the occurrence of a possible misalignment.

Moreover, there were samples such as S30 where certain variants were present only in the reference haplotype (namely 152C and 195C), and even though having a good variant strand bias (0.5 in both instances), their coverages were 24 and 23. More such examples can be found in S1, S2, S10, and S12 (where the variants in question, 16188Y and 16189Y, also had very low frequencies, 11.4 and 11.9). In context with the higher input and other variant coverages in the reference haplotype, these variants might be a result of low-level contamination and as such should not be interpreted.

## Conclusions

This study demonstrated that when attempting mtDNA haplotyping with low-quantity NGS libraries, which can result from badly preserved bones, more than half of the samples do match when comparing reference haplotype (30 pM, optimal library input) and suboptimal (0.3 pM) library input of the same sample. It also highlighted that alongside some true variants, lower library input might remove some variants that are possible result of low-level contamination. When presented with a forensic casework where only suboptimal quantity of sequencing libraries might be available, haplotypes obtained from mitogenome sequencing should be analyzed with caution.

### Supplementary Information

Below is the link to the electronic supplementary material.

Supplementary file1 (XLSX 11 KB)

Supplementary file2 (DOCX 18 KB)

Supplementary file3 (XLSX 85 KB)

## Data Availability

The authors declare that all the data are available.
