# Supplementary material for: Comparison of the optimal and suboptimal quantity of mitotype libraries using next-generation sequencing
Source: Int J Legal Med. 2023 Sep 30;138(2):395–400. doi: 10.1007/s00414-023-03099-7 (PMC10861744; doi:10.1007/s00414-023-03099-7)
Supplement: Supplementary file 2 — Supplementary file2 (DOCX 18 KB) [file 414_2023_3099_MOESM2_ESM.docx]

|  | Optimal library input (30 pM) – reference haplotypes | | | Suboptimal library input (0.3 pM) | | |
| --- | --- | --- | --- | --- | --- | --- |
| Sample name | **Mean Depth** | **Mapped Reads** | **Median Coverage** | **Mean Depth** | **Mapped Reads** | **Median Coverage** |
| S1 | 28708 | 322376 | 31814 | 493 | 5723 | 359 |
| S2 | 34729 | 398038 | 32859 | 529 | 6259 | 426 |
| S3 | 17394 | 199825 | 11447 | 282 | 3371 | 191 |
| S4 | 32651 | 367434 | 28025 | 571 | 6685 | 443 |
| S5 | 20348 | 229508 | 17146 | 410 | 4869 | 318 |
| S6 | 23912 | 268378 | 21245 | 471 | 5552 | 349 |
| S7 | 13795 | 160919 | 8594 | 240 | 2862 | 159 |
| S8 | 6270 | 71351 | 4347 | 131 | 1547 | 82 |
| S9 | 19398 | 220678 | 16261 | 300 | 3599 | 260 |
| S10 | 22316 | 297037 | 6873 | 467 | 6126 | 249 |
| S11 | 47296 | 525544 | 39619 | 709 | 8349 | 538 |
| S12 | 11162 | 127652 | 9560 | 165 | 1970 | 124 |
| S13 | 61199 | 688016 | 52496 | 888 | 10585 | 638 |
| S14 | 7296 | 79649 | 5131 | 100 | 1148 | 74 |
| S15 | 12464 | 139859 | 8147 | 241 | 2879 | 162 |
| S16 | 18238 | 202420 | 16739 | 272 | 3204 | 228 |
| S17 | 38812 | 442514 | 35385 | 712 | 8487 | 506 |
| S18 | 33896 | 376277 | 35946 | 634 | 7376 | 478 |
| S19 | 34015 | 383520 | 36464 | 628 | 7376 | 484 |
| S20 | 31811 | 359633 | 26959 | 696 | 8321 | 555 |
| S21 | 23781 | 269142 | 20596 | 457 | 5467 | 363 |
| S22 | 1332 | 25516 | 54 | 29 | 494 | 4 |
| S23 | 23 | 3213 | 6 | 0.264 | 25 | 0 |
| S32 | 11426 | 135234 | 3201 | 227 | 2776 | 155 |
| S25 | 7410 | 85677 | 2947 | 164 | 1985 | 100 |
| S26 | 19258 | 223375 | 9998 | 379 | 4566 | 273 |
| S27 | 17141 | 198851 | 10375 | 347 | 4146 | 222 |
| S28 | 18729 | 213987 | 15506 | 313 | 3744 | 245 |
| S29 | 20196 | 231473 | 20970 | 397 | 4767 | 302 |
| S30 | 11737 | 133703 | 2698 | 259 | 3067 | 175 |

SM3, Table 1: Mean depth, Mapped reads, and Median coverage at optimal (reference haplotypes) and suboptimal library input.
